# Supplementary material for: TRIM21 is a molecular rheostat for influenza A virus replication
Source: bioRxiv. 2026 Mar 17:2026.03.16.711963. Preprint. [Version 1] doi: 10.64898/2026.03.16.711963 (PMC13015713; doi:10.64898/2026.03.16.711963)
Supplement: Supplement 1 [file media-1.pdf]

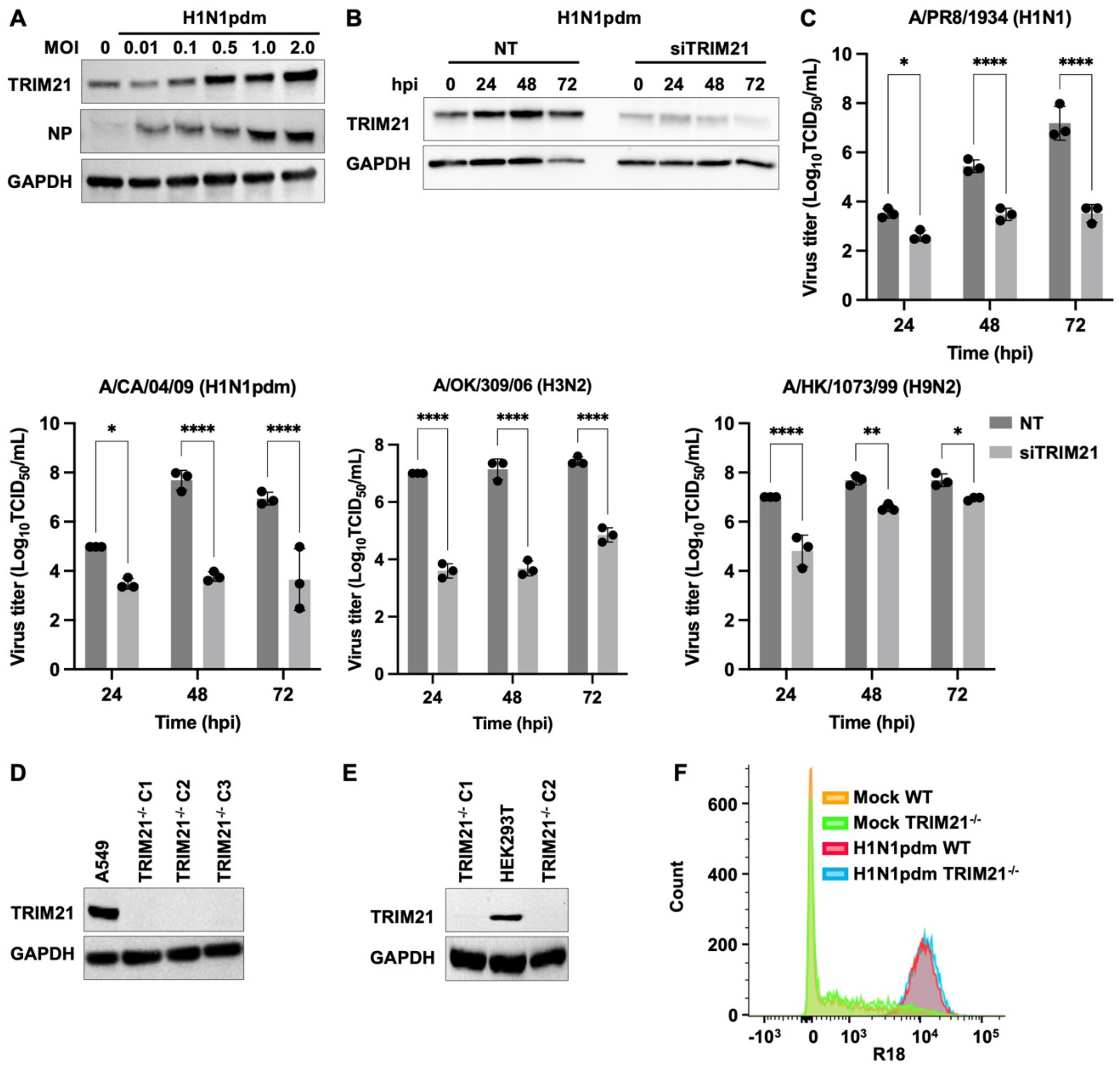

Figure S1. TRIM21 deficiency inhibits viral production. (A) A549 cells were infected with H1N1pdm at varying MOI (0.01, 0.1, 0.5, 1 and 2). Lysates prepared from infected cells were immunoblotted with antibodies against TRIM21 and NP. GAPDH was used as a loading control. **(B)** TRIM21 was depleted in A549 cells by siRNA-mediated knockdown. Non-targeting control (NT) and TRIM21-depleted (siTRIM21) cells were subsequently infected with H1N1pdm (MOI = 0.01). Knockdown efficiency was verified by immunoblotting using anti-TRIM21 antibody. **(C)** Viral titers in NT and siTRIM21 A549 cells infected with H1N1, H1N1pdm, H3N2, or H9N2 viruses (MOI = 0.01) at the indicated time points were measured by $TCID_{50/mL}$. Data are shown as means of n = 3 ± standard deviations (SD). Two-way ANOVA was used to analyze data (*p < 0.05, **p < 0.01, ****p < 0.0001). **(D and E)** Western blot validating CRISPR/Cas9-mediated TRIM21 deletion ($TRIM21^{-/-}$) in **(D)** A549 and **(E)** HEK293T cells. **(F)** Quantification of R18-positive population in mock and infected A549 cells, as measured by flow cytometry.

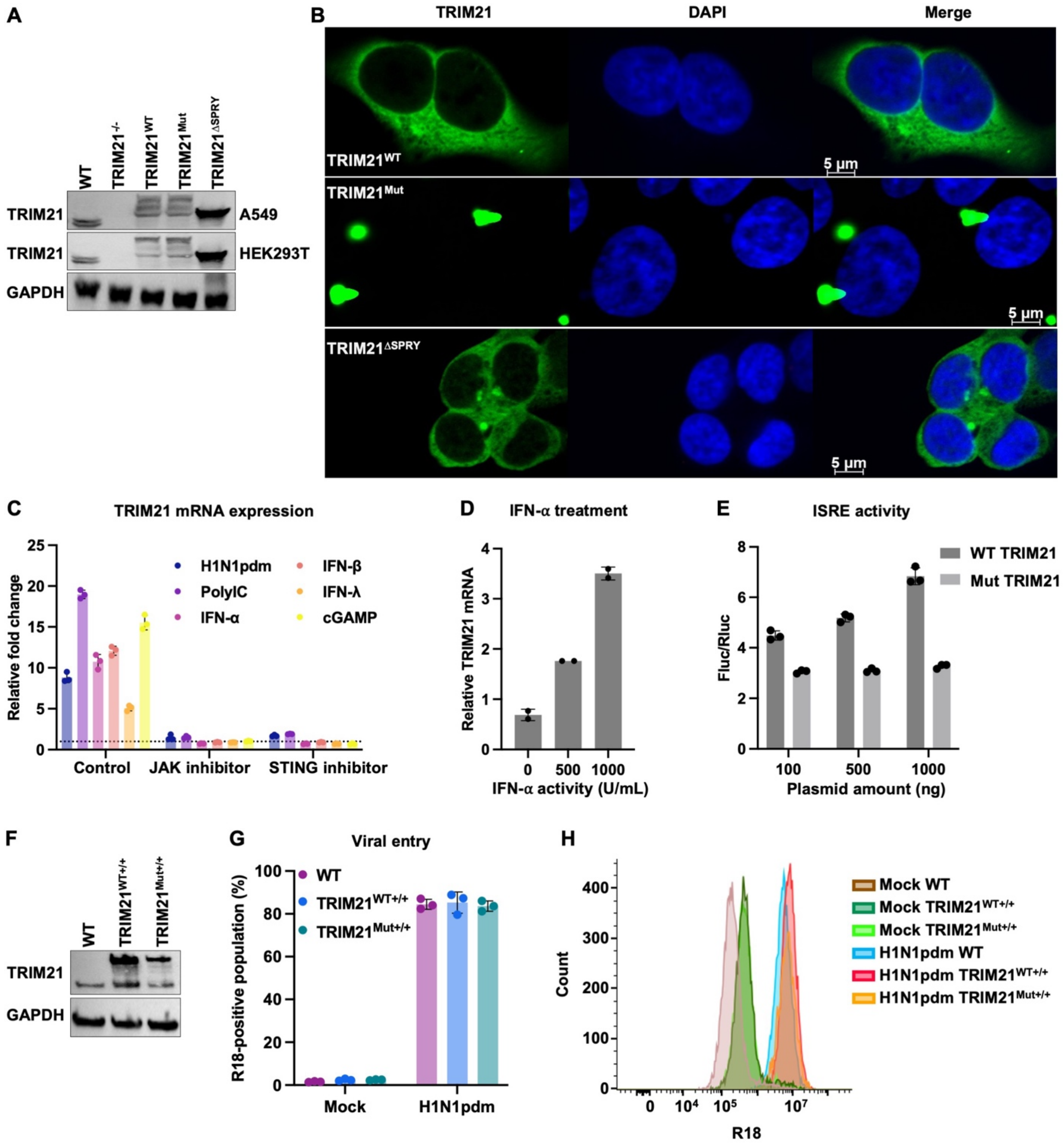

Figure S2. TRIM21 suppresses IAV replication by coupling NP degradation to innate immune amplification. (A) Immunoblot validation of A549 and HEK293T cells reconstituted with WT TRIM21 (TRIM21^{WT}), a catalytically inactive mutant of TRIM21 (TRIM21^{Mut}) or a SPRY domain-deficient TRIM21 (TRIM21$^{\Delta SPRY}$). TRIM21 constructs were GFP-tagged and therefore migrated at a higher molecular weight than endogenous TRIM21 (55 kDa). **(B)** Immunofluorescence imaging of TRIM21 subcellular localization in the indicated reconstituted A549 cell lines. **(C)** A549 cells were treated with the indicated innate immune stimuli in the presence or absence of pathway-specific inhibitors. Total RNA was isolated, and TRIM21 mRNA levels were quantified by real-time PCR. Data are shown as means of n = 3 ± standard deviations (SD). The dotted line indicates fold change of 1. **(D)** A549 cells were treated with increasing concentrations of IFN-α, followed by analysis of TRIM21 mRNA expression by real-time PCR.

Data are shown as means of n = 2 ± standard deviations (SD). **(E)** HEK293T cells were transiently transfected with increasing amounts of plasmids expressing WT Myc-TRIM21 or the catalytically inactive mutant Myc-TRIM21, together with an ISRE-driven firefly luciferase reporter and a Renilla luciferase control. At 24 hours post-transfection, cells were treated with IFN-α for an additional 24 hours prior to measurement of luciferase activities. Data are shown as means of n = 3 ± standard deviations (SD). **(F)** Immunoblot validation of A549 cells stably overexpressing WT TRIM21(TRIM21$^{WT+/+}$) or the catalytically inactive mutant (TRIM21$^{Mut+/+}$). TRIM21 constructs were GFP-tagged and therefore migrated at a higher molecular weight than endogenous TRIM21 (55 kDa). **(G and H)** Viral entry was assessed by flow cytometric analysis of R18-positive populations at 1 hpi. Data are shown as means of n = 3 ± standard deviations (SD).

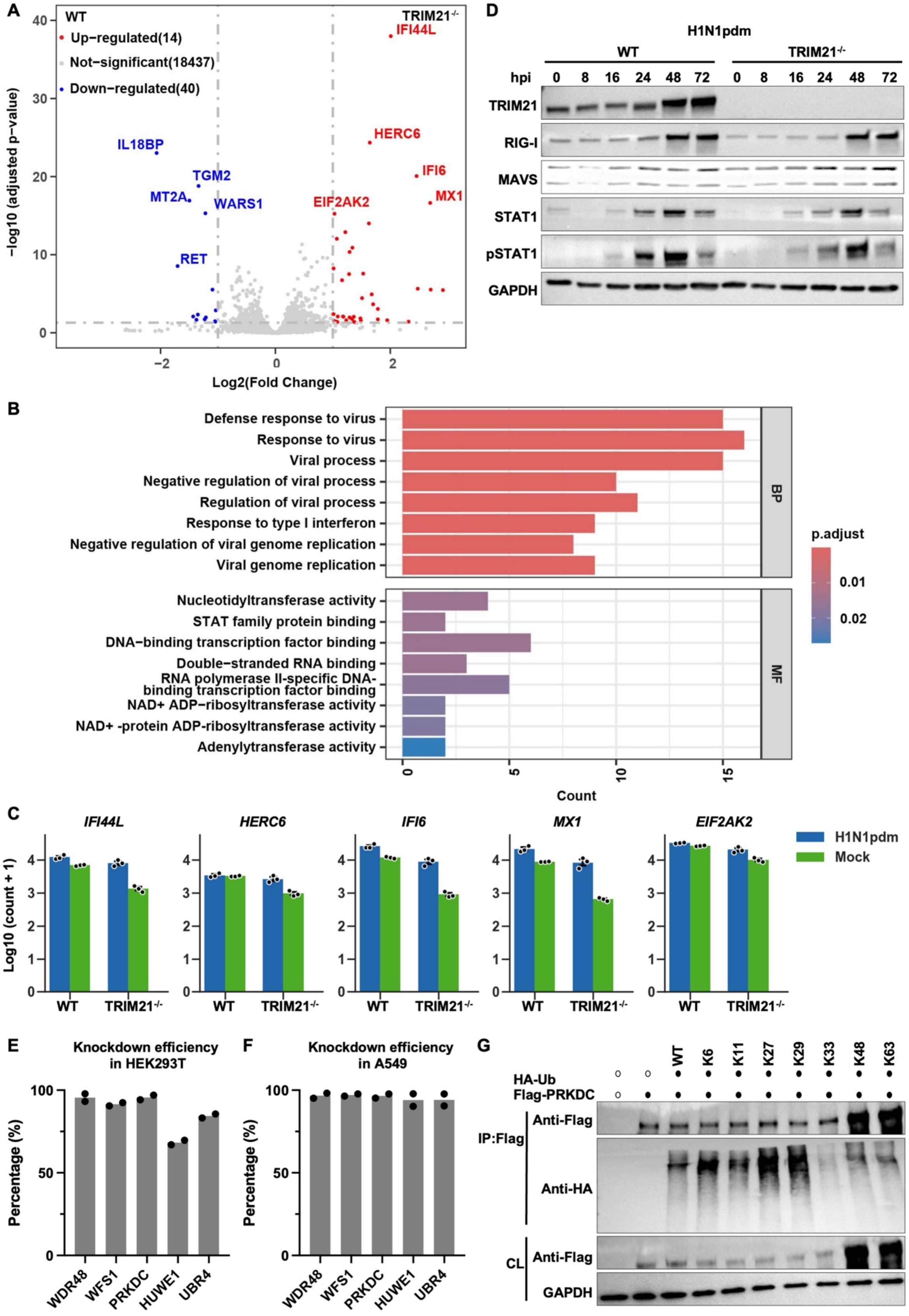
A
WT
TRIM21-/-
Up−regulated(14)
Not−significant(18437)
Down−regulated(40)
IFI44L
HERC6
IFI6
MX1
EIF2AK2
IL18BP
TGM2
MT2A
WARS1
RET
−log10 (adjusted p−value)
Log2(Fold Change)
B
Defense response to virus
Response to virus
Viral process
Negative regulation of viral process
Regulation of viral process
Response to type I interferon
Negative regulation of viral genome replication
Viral genome replication
Nucleotidyltransferase activity
STAT family protein binding
DNA−binding transcription factor binding
Double−stranded RNA binding
RNA polymerase II-specific DNA-binding transcription factor binding
NAD+ ADP−ribosyltransferase activity
NAD+ -protein ADP-ribosyltransferase activity
Adenylytransferase activity
BP
MF
p.adjust
Count
C
IFI44L
HERC6
IFI6
MX1
EIF2AK2
H1N1pdm
Mock
Log10 (count + 1)
WT
TRIM21-/-
D
H1N1pdm
WT
TRIM21-/-
hpi
TRIM21
RIG-I
MAVS
STAT1
pSTAT1
GAPDH
E
Knockdown efficiency in HEK293T
F
Knockdown efficiency in A549
Percentage (%)
WDR48
WFS1
PRKDC
HUWE1
UBR4
G
HA-Ub
Flag-PRKDC
WT
K6
K11
K27
K29
K33
K48
K63
IP:Flag
Anti-Flag
Anti-HA
CL
GAPDH

Figure S3. Loss of TRIM21 reveals a PRKDC-dependent compensatory antiviral program. **(A)** Volcano plot showing differentially expressed genes (DEGs) between H1N1pdm-infected WT and TRIM21$^{-/-}$ A549 cells (MOI 0.01, 48hpi). The x-axis indicates $\log_2$ fold change and the y-axis shows $-\log_{10}$ adjusted *p*-value. DEGs were defined using an adjusted *p*-value cutoff of 0.05 and an absolute $\log_2$ fold change cutoff of 1. Colors indicate upregulated (red) or downregulated (blue) genes. **(B)** Gene Ontology analysis for upregulated DEG in biological process (BP) and molecular function (MF) in H1N1pdm-infected TRIM21$^{-/-}$ A549 cells. The number on the x-axis indicates the enriched count of DEG. **(C)** Transcriptomic raw count of ISGs in mock- and H1N1pdm-infected WT and TRIM21$^{-/-}$ A549 cells (MOI 0.01, 48hpi). **(D)** WT and TRIM21$^{-/-}$ A549 cells were infected with H1M1pdm (MOI = 0.01), and cell lysates were immunoblotted for TRIM21, RIG-I, MAVS, STAT1, and phosphorylated (pSTAT1). **(E and F)** siRNA-mediated knockdown efficiency of the indicated targets in **(E)** HEK293T and **(F)** A549 cells measured by real-time PCR. **(G)** HEK293T cells were transiently co-transfected with Flag-PRKDC and HA-ubiquitin (WT versus linkage-specific constructs as indicated). Cells were treated with MG132, followed by immunoprecipitation using anti-Flag M2 affinity beads. IP and CL were then analyzed by immunoblotting. Black and white dots indicate the presence or absence of plasmid transfection, respectively.

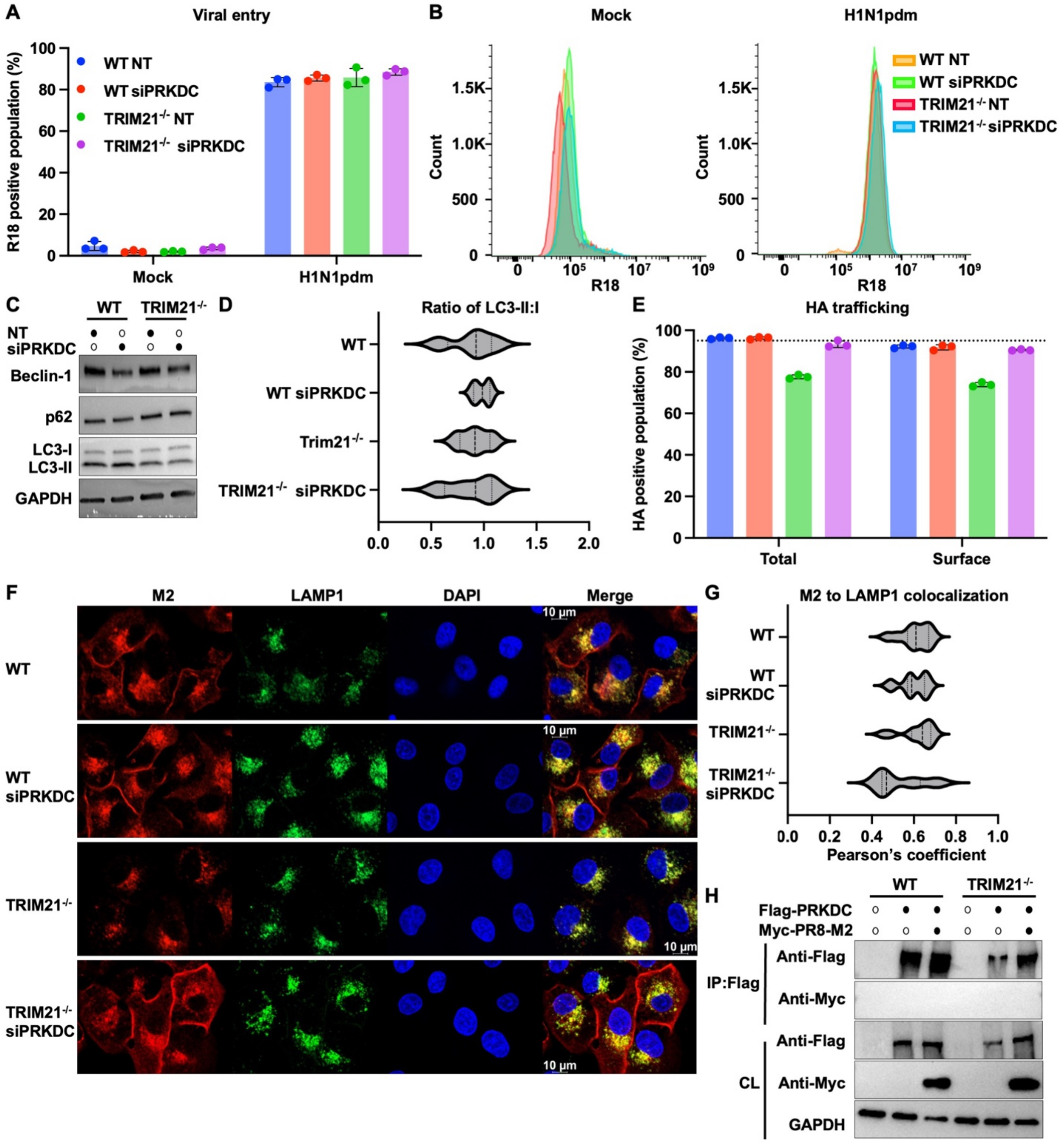

Figure S4. PRKDC-mediated restriction is independent of viral entry and autophagic degradation of viral proteins. (A and B) Viral entry was assessed by flow cytometric analysis of R18-positive populations at 1 hpi. Data are shown as means of n = 3 ± standard deviations (SD). **(C)** WT and TRIM21$^{-/-}$ A549 cells were transfected with NT or PRKDC-targeting siRNA and subsequently infected with H1N1pdm (MOI = 0.01). Lysates were immunoblotted Beclin-1, p62, and LC3. The cell lines are indicated by black dots. **(D)** Ratio of LC3-II to LC3-I were quantified by densitometry. Each violin represents *n*= 4 independent samples. **(E)** WT and TRIM21$^{-/-}$ A549 cells were transfected with NT or PRKDC-targeting siRNA and subsequently infected with H1N1pdm (MOI = 10). Total and surface expression of viral HA protein were quantified by flow cytometry. The dotted line indicates the mean HA expression in

NT-treated WT A549 cells. Data are shown as means of n = 3 ± SD. **(F)** Immunofluorescence imaging of viral M2 and the lysosomal marker LAMP1 were performed in indicated H1N1pdm-infected A549 cells (MOI = 10). (**G)** Colocalization coefficient between M2 and LAMP1 in the indicated H1N1pdm infected A549 cells was quantified. Each violin represents *n* = 11 images, except for TRIM21$^{-/-}$ siPRKDC (*n* = 10). **(H)** HEK293T cells were transiently co-transfected with Flag-PRKDC and Myc-PR8-M2. Lysates were immunoprecipitated with anti-Flag M2 affinity beads and analyzed by immunoblotting. Black and white dots indicate the presence or absence of plasmid transfection, respectively.

A Positive selection analysis

Mammalian order		Starting omega 0.4	Starting omega 1.5
Primates	Full	0.09	0.09
	SPRY	0.21	0.21
Rodents	Full	0.02	0.21
	SPRY	0.31	0.31
Carnivores	Full	0.19	0.19
	SPRY	0.54	0.19
Even-toed ungulates	Full	0.24	0.24
	SPRY	0.99	0.99

B Multiple sequence alignment of TRIM21 RING domain

```
Human        CPICLDPFVEPVSIECGHSFCQECI
Chimpanzee   CPICLDPFVEPVSIECGHSFCQECI
Macaque      CPICLDPFVEPVSIECGHSFCQECI
Mouse        CSICLDPMVEPMSIECGHCFCKECI
Rat          CSICLEPMVEPMSIECGHCFCKECI
Ferret       CPICLDPMVEPMSIDCGHNFCHECI
Hamster      CSICLDPMVAPMSIECGHCFCKECI
Pig          CSICLDPMVEPVSIECGHSFCQECI
Seal         CPICLDPMVEPMSIDCGHSFCHECI
```

C Multiple sequence alignment of TRIM21 SPRY domain

```
Human        ELRSVCHVPGLKKMLRTCAVHITLDPDTANPWLILSEDRRQVRLGDTQQSIPGNEERFDSYPMVLGAQHFHSG
Chimpanzee   ELRSVCHVPGLKKMLRTCAVHITLDPDTANPWLILSEDRRQVRLGDTQQSIPGNEERFDSYPMVLGAQHFHSG
Macaque      ELRSVCHVPGLKKMLRTCAVHITLDPDTANPWLILSEDRRQVRLGDTQQSIPGNEERFDSYPMVLGAQHFHSG
Mouse        DLTSTCPVPGRKKMLRTCWVHITLDRNTANSWLIISKDRRQVRMGDTHQNVSDNEERFSNYPMVLGAQRFSSG
Rat          DLTSTCHVPGRKKMLRTCWVHITLDRDTANSCLIISKDRRQVRMGDTYQNVSENAERFNNYPMVLGAQRFSSG
Ferret       DLRNVCLVPGLKRMLRTCGVHITLDPHTANPWLILSEDRRQVRLGDRRQEVPENEDRFDHYPMVLGAQCFDSG
Hamster      DLTNACHVPGRKKMLRTCWVHITLDRNTANPWLIIPKDRRQVRIGDSHQNVSENDERFNNYPVVLGAQRFTSG
Pig          DLSILCYVPGIKRMLRSYAVHITLDPLTASPWLVLSENRRQVRLGETRQEVPENEERFDTYPMVLGAQHFNSG
Seal         DLRSVCLVPGLKRMLRTCGVHITLDPHTANPWLILSKDQRQVRLGDSQQKVPENEDRFDSYPMVLGAQCFDSG

Human        KHYWEVDVTGKEAWDLGVCRDSVRRKGHFLLSSKSGFWTIWLWNKQKYEAGTYPQTPLHLQVPPCQVGIFLDY
Chimpanzee   KHYWEVDVTGKEAWDLGVCRDSVRRKGHFLLSSKSGFWTIWLWNKQKYEAGTYPQTPLHLQVPPCQVGIFLDY
Macaque      KHYWEVDVTGKEAWDLGVCRDSVRRKGHFLLSSKSGFWTIWLWNKQKYEAGTYPQTPLHLQVPPCQIGIFLDY
Mouse        KMYWEVDVTQKEAWDLGVCRDSVQRKGQFSLSPENGFWTIWLW-QDSYEAGTSPQTTLHIQVPPCQIGIFVDY
Rat          KMYWEVDVTQKQAWDLGVCRDSVQRKGHFSLSPENGFWTIWLWQKDNYEAGTSPQTTLHIQVPPCQIGIFVDY
Ferret       QVYWEVDVTGKEAWDLGVCRDSVQRKGQFSLSPETGFWTIWLWNKQKYEAGTCIQTPLHLQVPPRQVGIFLDY
Hamster      KMYWEVDVTRKEAWELGICRESVQRKGQFLFSPENGFWIIWLWNKDIYEAGTNPQTTLHLQVPPCQVGIFVDY
Pig          KHYWEVDVAGKEAWDLGVCRDSVKRKGHFLLNPSNGFWTIWLWNKQKYEAGTCTQTPLYPQVPPSRVGIFLDY
Seal         KVYWEVDVTGKEAWDLGVCRDSVQRKGHFLLSPENGFWTIWLWNKQKYEAGTCPQTPLHLQVPPRQVGIFLDC

Human        EAGMVSFYNITDHGSLIYSFSECAFTGPLRPFFSPGFNDGGKNTAPLTLCPL
Chimpanzee   EAGMVSFYNITDHGSLIYSFSECAFTGPLRPFFSPGFNDGGKNTAPLTLCPL
Macaque      EAGMVSFYNITDHGSLIYSFSECAFTGPLRPFFSPGFNDGGRNTAPLTLCPL
Mouse        EAGVVSFYNITDHGSLIYTFSECVFAGPLRPFFNVGFNYSGGNAAPLKLCPL
Rat          EAGIVSFYNITDHGSLIYTFSECAFIGPLRPFFSVGFNDDGGNSAPLKLCPL
Ferret       EARIVSFYNITDHGSLIYTFSECAFAEPLRPFFSTGFNDGGRNAAPLVLCPL
Hamster      EAGIVSFYNITDHGSLIYTFSQCTFAGPLRPFFSVGFNDDGGNAAPLKLCPL
Pig          DASTVSFYNISDHGSLIYSFSECAFAGPVRPFFNPGFNDGGRNAPPLTLCPL
Seal         EARIVSFYNVTDHGSLIYTFSECAFAGPLRPFFSTGFNDEGRNGAPLVLCPL
```

Figure S5. Comparative evolutionary analysis of TRIM21 across mammals. (A) Positive selection analysis of full-length and SPRY domain of TRIM21 in the indicated mammalian order. Analyses were performed using orthologous TRIM21 sequences listed in Table S2. **(B and C)** Multiple sequence alignment of TRIM21 from representative species highlighting conservation within the **(B)** RING domain and **(C)** SPRY domain. Catalytic residues and residues implicated in NP binding are marked with red.
